# Prognostic significance of albumin-to-globulin ratio in patients with renal cell carcinoma: a meta-analysis

**DOI:** 10.3389/fonc.2023.1210451

**Published:** 2023-07-19

**Authors:** Huaying Mao, Fan Yang

**Affiliations:** ^1^ Clinical Laboratory, Huzhou Central Hospital, Affiliated Central Hospital of Huzhou University, Huzhou, Zhejiang, China; ^2^ Clinical Laboratory, Huzhou Maternity and Child Health Care Hospital, Huzhou, Zhejiang, China

**Keywords:** albumin-to-globulin ratio, renal cell carcinoma, meta-analysis, prognosis, blood test

## Abstract

**Background:**

Whether the albumin-to-globulin ratio (AGR) predicts the prognosis of renal cell carcinoma (RCC) remains controversial. Herein, we performed a meta-analysis to critically evaluate the relationship between the AGR and RCC prognosis, as well as the association between the AGR and the clinicopathological characteristics of RCC.

**Methods:**

The PubMed, Web of Science, Embase, and Cochrane Library databases were thoroughly and comprehensively searched from their inception until 24 June 2023. To determine the predictive significance of the AGR, hazard ratios (HRs) and corresponding 95% confidence intervals (CIs) were calculated from the pooled data. The relationship between the AGR and the clinicopathological features of RCC was evaluated by estimating odds ratios (ORs) and 95% CIs in subgroup analyses.

**Results:**

The meta-analysis included nine articles involving 5,671 RCC cases. A low AGR significantly correlated with worse overall survival (OS) (HR = 1.82, 95% CI = 1.37–2.41, p <0.001) and progression-free survival (PFS) (HR = 2.44, 95% CI = 1.61–3.70, p <0.001). Analysis of the pooled data also revealed significant associations between a low AGR and the following: female sex (OR = 1.48, 95% CI = 1.31–1.67, p <0.001), pT stage T3–T4 (OR = 4.12, 95% CI = 2.93–5.79, p <0.001), pN stage N1 (OR = 3.99, 95% CI = 2.40–6.64, p <0.001), tumor necrosis (OR = 3.83, 95% CI = 2.23–6.59, p <0.001), and Fuhrman grade 3–4 (OR = 1.82, 95% CI = 1.34–2.42, p <0.001). The AGR was not related to histology (OR = 0.83, 95% CI = 0.60–1.15, p = 0.267).

**Conclusion:**

In patients with RCC, a low AGR strongly predicted poor OS and PFS and significantly correlated with clinicopathological features indicative of disease progression.

## Introduction

Renal cell carcinoma (RCC) accounts for approximately 2.2% of all cancer cases, and the median age at diagnosis age is 64 years (1, 2). Most (approximately 90%) malignant solid lesions in the kidney are RCCs (3). Globally, there were 431,288 new cases of RCC and 179,368 deaths from RCC in 2020 (1). Since 2012, the number of people developing RCC worldwide has increased by 22% according to the World Cancer Research Fund International (4).

RCC has three major subtypes: clear cell (approximately 80% of cases), papillary (approximately 15%), and chromophobe (approximately 5%) (5, 6). Despite significant advances in therapeutic strategies, RCC still has a poor prognosis. The overall 5-year survival rate is 8–59% (7); for advanced disease, it is <20%, and for metastatic RCC (mRCC), the median overall survival (OS) time is 10 months (8, 9). These poor outcomes may partly reflect the lack of powerful prognostic indicators (10). Consequently, identifying novel and effective biomarkers for prognosis prediction in patients with RCC in clinical settings is imperative.

Current evidence indicates that systemic chronic inflammation and malnutrition contribute to carcinogenesis and tumor progression (11, 12). Albumin (ALB) and globulin (GLB) are two major serum proteins that reflect nutritional and inflammatory status. The ALB-to-GLB ratio (AGR) is an established marker in oncology; it is calculated as follows: AGR = serum ALB/(total serum protein - serum ALB) (13). A low AGR has been widely associated with poor outcomes in various cancers, such as non-small-cell lung cancer (14), esophageal cancer (15), cervical cancer (16), multiple myeloma (17), and pancreatic cancer (18).

Previous studies have explored the prognostic significance of the AGR in patients with RCC (19–27). However, inconsistent results were obtained: in some studies, a low AGR significantly predicted worse survival (23, 26, 27), whereas in others, the AGR was unrelated to prognosis (21, 24, 25). To provide resolution, we performed a meta-analysis that evaluated the relationship between the AGR and RCC prognosis, as well as between the AGR and the clinicopathological characteristics of RCC.

## Materials and methods

### Study guideline

This meta-analysis was performed in accordance with the Preferred Reporting Items for Systematic Reviews and Meta-Analyses guidelines (28).

### Ethics statement

This study used data from previous articles, and approval from an ethics committee or institutional review board was therefore waived.

### Search strategy

We thoroughly and comprehensively searched the PubMed, Web of Science, Embase, and Cochrane Library databases from their inception until 24 June 2023. Only English publications were selected. The following combinations of Medical Subject Headings and other terms were used: (albumin to globulin ratio, albumin/globulin ratio, AGR, or albumin-globulin ratio) and (renal cancer, kidney neoplasm, kidney cancer, renal cell carcinoma, renal carcinoma, or RCC). The references of the retrieved studies were manually examined to identify additional relevant studies.

### Study eligibility criteria

We included English-language articles that reported the following: (1) the association between the AGR and survival outcome [e.g., OS, recurrence-free survival, cancer-specific survival (CSS), and progression-free survival (PFS)]; (2) hazard ratios (HRs) with 95% confidence intervals (CIs) or data that allowed their calculation; and (3) the threshold used to stratify a high/low AGR. Additional inclusion criteria were a pathological diagnosis of RCC and measurement of serum ALB and GLB levels before treatment. Meeting abstracts, reviews, case reports, letters, comments, studies with overlapping patients, and animal studies were excluded.

### Data collection and quality evaluation

Two researchers (HM and FY) independently screened the retrieved articles and extracted and crosschecked the data. Disagreements between the two investigators were settled through negotiation until a consensus was reached. The data collected in this study included first author, country, publication year, sample size, study period, patient age and sex, number of study centers (single or multiple), metastatic status, treatment, follow-up period, AGR threshold, survival endpoints, type of survival analysis (univariate or multivariate), and HRs with 95% CIs. We used the Newcastle-Ottawa Scale (NOS) (29) to evaluate the quality of the study in three domains: selection (0–4 points), comparability (0–2 points), and outcome assessment (0–3 points), yielding a total score of 0–9. Articles with NOS scores ≥6 were considered high-quality.

### Statistical analysis

HRs with 95% CIs were calculated to determine whether the AGR significantly predicted survival outcome. Inter-study heterogeneity was assessed using Higgin’s I^2^ statistic (30) and Cochran’s Q test (31). P values <0.10 and I^2^ values >50% indicated heterogeneity. Random-effects and fixed-effects models are used for heterogeneous and non-heterogeneous data, respectively.

To further explore the ability of the AGR to predict RCC prognosis, subgroup analyses stratified by country, sample size, study center number, metastatic status, treatment, AGR threshold, and type of survival analysis were performed. Associations between the AGR and the clinicopathological features of RCC were evaluated by determining odds ratios (ORs) with 95% CIs. Begg’s test (32) and Egger’s test (33) were used to assess publication bias. Statistical analyses were performed using Stata software, version 12.0 (Stata Corporation, College Station, TX, USA). P values <0.05 indicated statistical significance.

## Results

### The literature selection process

**Figure 1:**
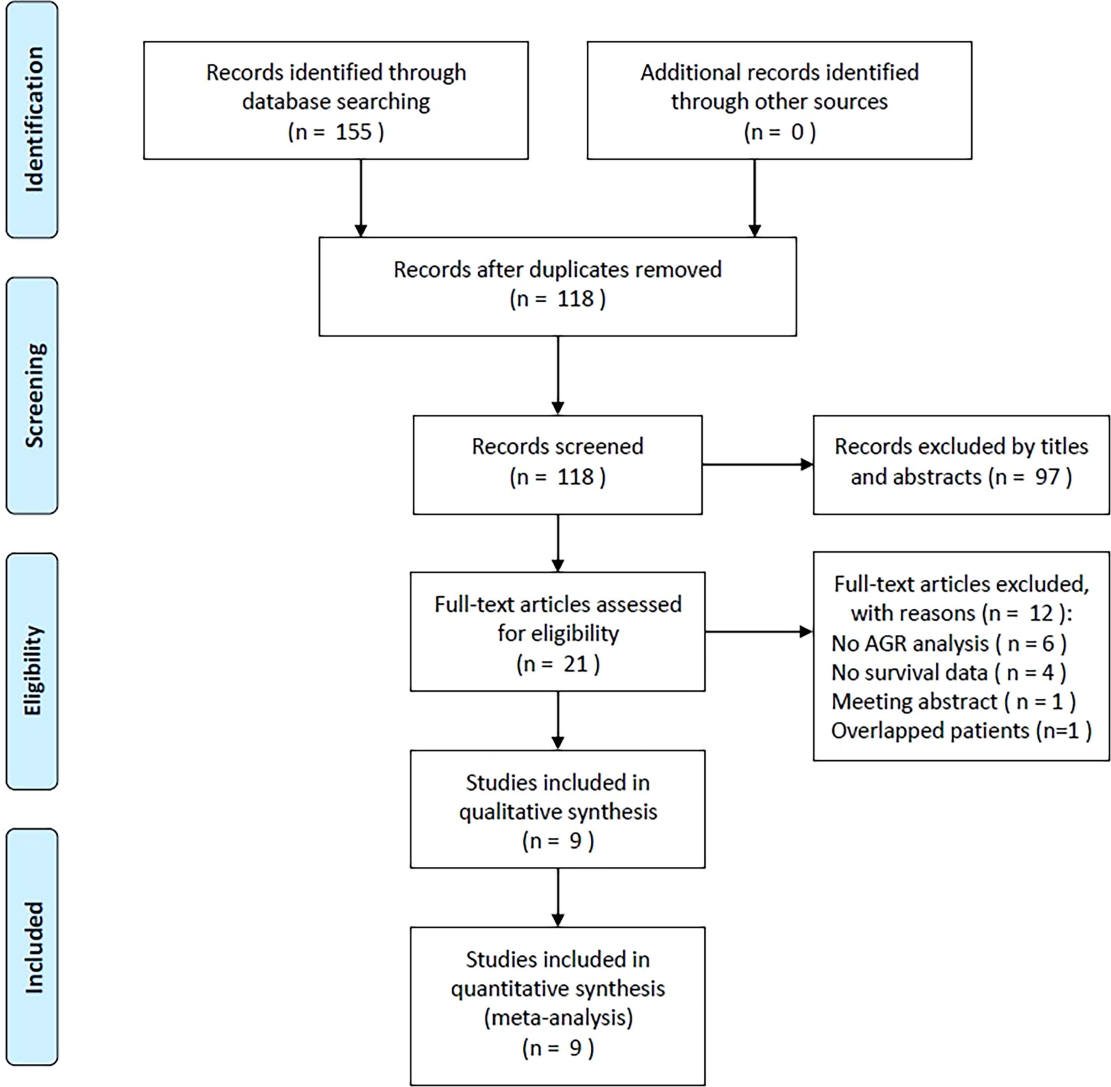
PRISMA flowchart of the established screening strategy.

A total of 155 studies were retrieved in the preliminary search; 37 duplicates were eliminated, leaving 118 studies (**Figure 1**). An additional 97 studies were excluded owing to irrelevance as determined upon title and abstract screening. Among the remaining 21 studies, 12 were discarded following full-text assessment: six did not analyze AGRs, four lacked survival data, one was a meeting abstract, and one had overlapping patients. Finally, nine articles involving 5,671 cases (19–27) were included in the present study.

### Study features

**Table 1** lists the basic features of the nine included studies. All studies were published between 2017 and 2021; five were performed in China (19, 20, 22, 24, 25), two in Turkey (21, 26), and one each in Korea (23) and Austria (27). All studies were retrospective (19–27), with sample sizes of 95–2,970 (median, 187). Seven studies were single-center (19–22, 24–26) and two were multicenter (23, 27). Four studies examined patients with non-mRCC (19, 21, 23, 24), three examined patients with mRCC (25–27), and two included patients at multiple stages (20, 22). Surgery was performed in eight studies (19–25, 27) and targeted therapy in one study (26). The threshold AGR was 1.11–1.64 (median, 1.43). Eight (19–21, 23–27) and six (19, 21–23, 26, 27) studies assessed the significance of the AGR in predicting OS and PFS, respectively. Multivariate regression analyses with HRs and CIs were performed in seven studies (19–21, 23, 24, 26, 27) and univariate analyses in two (22, 25). The NOS scores for the included articles ranged from 7 to 9, which indicated high quality.

**Table 1:** Baseline characteristics of included studies in this meta-analysis.

Study	Year	Country	Sample size	Study period	Age (year) Median (range)	Study center	Gender (M/F)	Study design	Metastatic status	Treatment	Cut-off value	Follow-up (month) Median (range)	Survival outcomes	Survival analysis	NOS score
Chen, Z.	2017	China	416	2003-2013	56.3 (24-80)	Single center	258/158	Retrospective	Non-metastatic	Surgery	1.22	69.2 (1-151)	OS, PFS	Multivariate	7
He, X.	2017	China	895	2000-2012	51.4	Single center	600/295	Retrospective	Mixed	Surgery	1.47	69.68	OS	Multivariate	7
Koparal, M. Y.	2018	Turkey	162	2010-2016	56.5	Single center	102/60	Retrospective	Non-metastatic	Surgery	1.40	27.5 (6-89)	OS, PFS	Multivariate	8
Bian, Z.	2020	China	187	2011-2017	56.7	Single center	118/69	Retrospective	Mixed	Surgery	1.64	54.6 (1-97.2)	PFS	Univariate	7
Chung, J. W.	2020	Korea	2,970	1999-2015	55.6	Multicenter	2,055/915	Retrospective	Non-metastatic	Surgery	1.47	26.0 (9.0-59.0)	OS, PFS	Multivariate	9
Hu, J.	2020	China	170	2010-2015	52.5	Single center	120/50	Retrospective	Non-metastatic	Surgery	1.35	70	OS	Multivariate	7
Xu, K.	2020	China	95	2005-2016	56 (16-75)	Single center	84/11	Retrospective	Metastatic	Surgery	1.5	51 (6-132)	OS	Univariate	8
Aktepe, O. H.	2021	Turkey	163	2008-2019	60 (53-65)	Single center	120/43	Retrospective	Metastatic	Targeted therapy	1.11	19.05 (1.31-102.6)	OS, PFS	Multivariate	7
Laukhtina, E.	2021	Austria	613	NR	57 (50-64)	Multicenter	428/185	Retrospective	Metastatic	Surgery	1.43	31	OS, PFS	Multivariate	8

M, male; F, female; OS, overall survival; PFS ,progression-free survival; NOS, Newcastle-Ottawa Scale.

### AGR and OS

**Figure 2:**
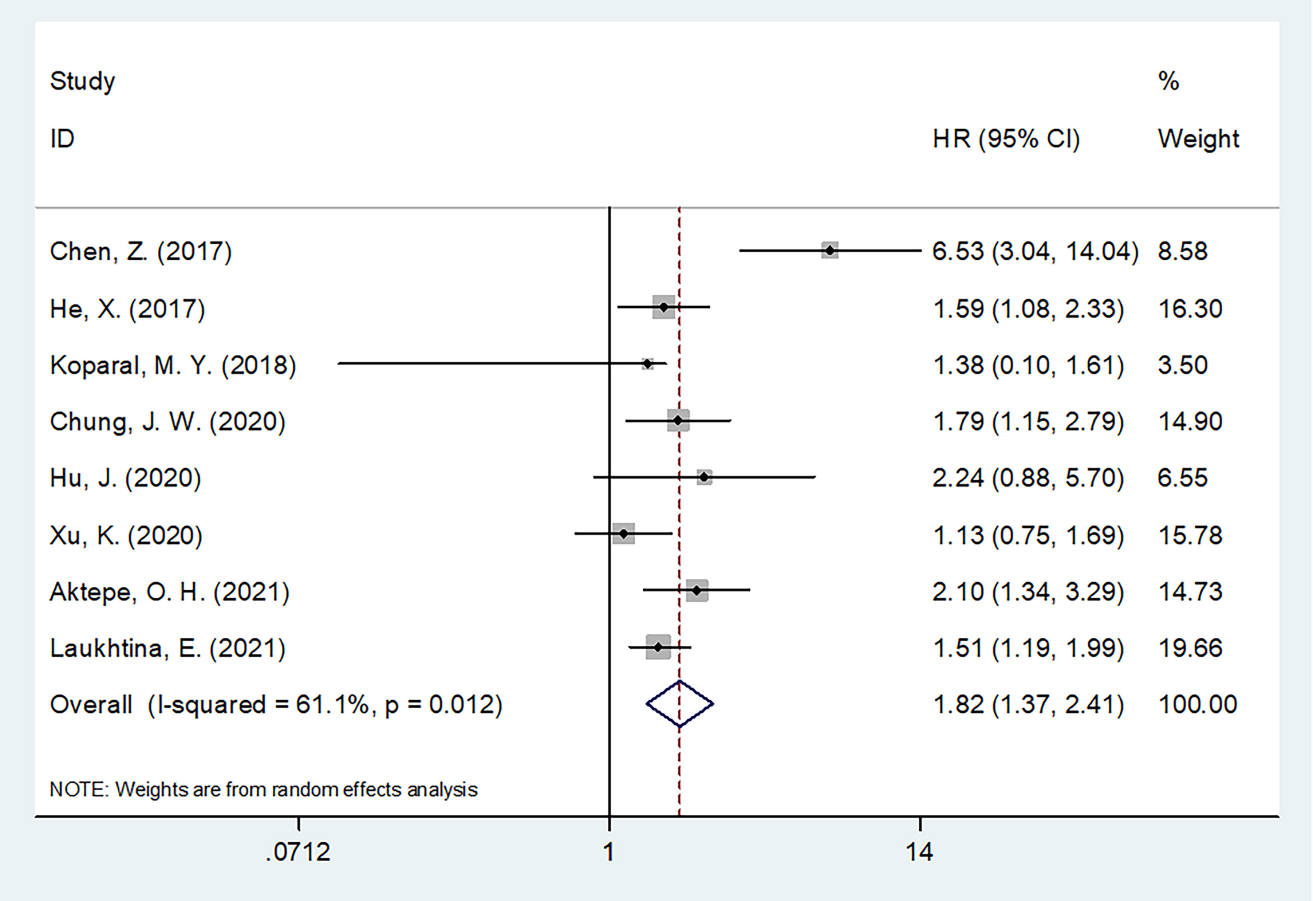
Forest plots of the prognostic role of AGR for OS in patients with RCC.

Eight studies with 5,484 patients provided information regarding the relationship between the pretreatment AGR and OS (19–21, 23–27). Owing to obvious heterogeneity (I^2^ = 61.1%, p = 0.012), we used a random-effects model to collectively analyze the data in these studies. We found that a low AGR remarkably predicted worse OS (HR = 1.82, 95% CI = 1.37–2.41, p <0.001; **Table 2** and **Figure 2**). In subgroup analyses, the ability of the AGR to predict OS was unaffected by sample size, country, study center number, metastatic status, treatment, or AGR threshold (**Table 2**). Moreover, a low AGR significantly predicted poor OS in a multivariate analysis (HR = 1.98, 95% CI = 1.47–2.66, p <0.001; **Table 2**).

**Table 2:** Subgroup analysis of prognostic value of AGR for OS in patients with RCC.

Variables	No. of studies	No. of patients	Effects model	HR (95%CI)	p	Heterogeneity I^2^(%) Ph	
Total	8	5,484	Random	1.82 (1.37-2.41)	<0.001	61.1	0.012
Country							
China	4	1,576	Random	2.11 (1.10-4.05)	0.025	81.5	0.001
Other than China	4	3,908	Fixed	1.66 (1.37-2.02)	<0.001	0	0.623
Sample size							
<200	4	590	Fixed	1.54 (1.16-2.04)	0.002	37.0	0.190
≥200	4	4,894	Random	2.04 (1.32-3.14)	0.001	76.7	0.005
Study center							
Single center	6	1,901	Random	1.99 (1.27-3.13)	0.003	70.8	0.004
Multicenter	2	3,583	Fixed	1.58 (1.26-1.97)	<0.001	0	0.511
Metastatic status							
Non-metastatic	4	3,718	Random	2.57 (1.28-5.14)	0.008	66.2	0.031
Metastatic	3	871	Random	1.51 (1.12-2.04)	0.007	51.0	0.130
Mixed	1	895	–	1.59 (1.08-2.33)	0.019	–	–
Treatment							
Surgery	7	5,321	Random	1.78 (1.29-2.46)	<0.001	64.3	0.010
Targeted therapy	1	163	–	2.10 (1.34-3.29)	0.001	–	–
Cut-off value							
<1.43	4	911	Random	2.73 (1.45-5.12)	0.002	59.0	0.062
≥1.43	4	4,573	Fixed	1.48 (1.25-1.77)	<0.001	0	0.456
Survival analysis							
Univariate	1	95	–	1.13 (0.75-1.69)	0.567	–	–
Multivariate	7	5,389	Random	1.98 (1.47-2.66)	<0.001	56.7	0.031

### AGR and PFS

**Figure 3:**
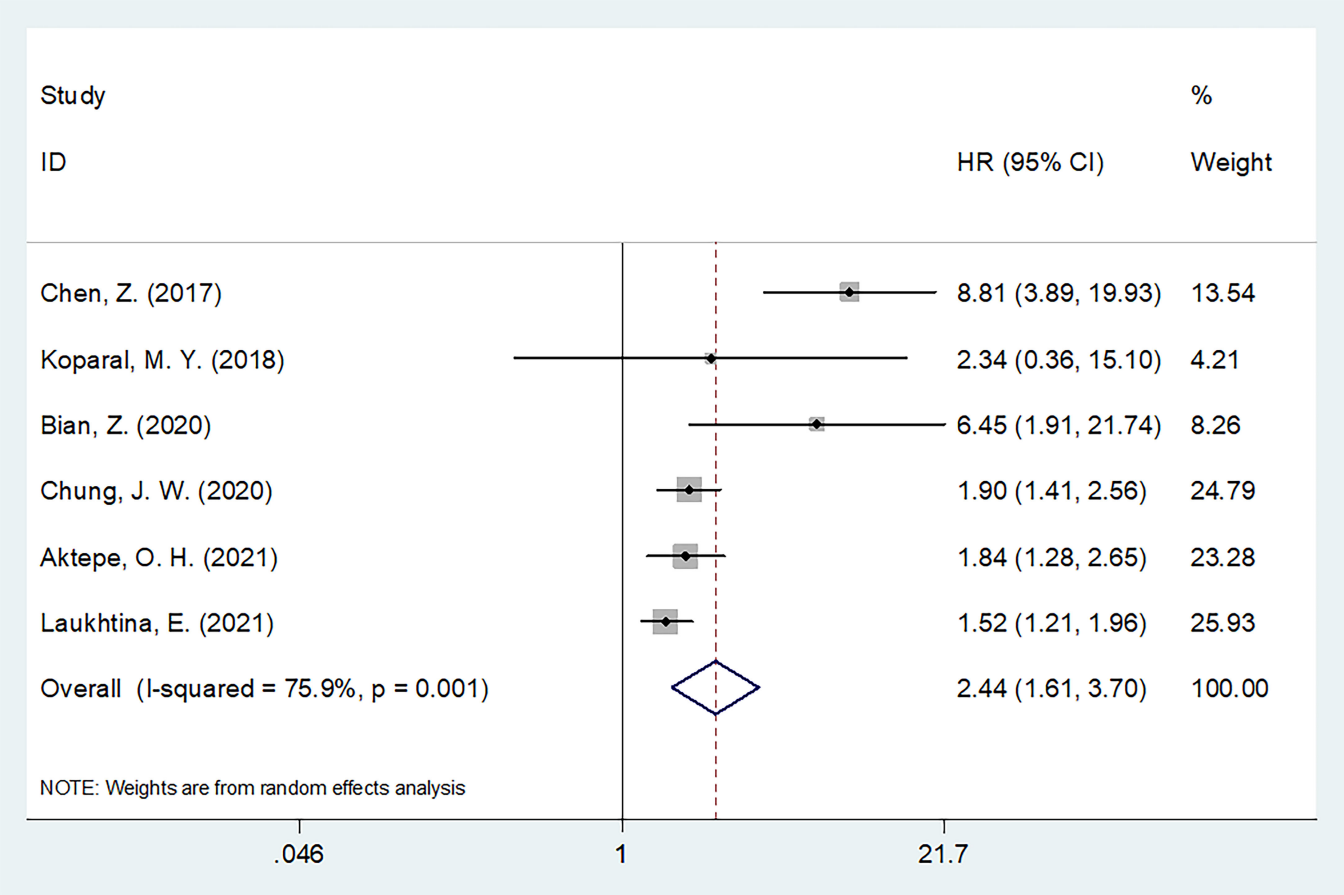
Forest plots of the prognostic role of AGR for PFS in patients with RCC.

Six articles with 4,511 patients examined the relationship between the pretreatment AGR and PFS (19, 21–23, 26, 27). Owing to obvious heterogeneity (I^2^ = 75.9%, p = 0.001), we used a random-effects model to collectively analyze the data in these articles. We found that a low AGR strongly predicted worse PFS (HR = 2.44, 95% CI = 1.61–3.70, p <0.001; **Figure 3** and **Table 3**). In subgroup analyses, a low AGR significantly predicted poor PFS regardless of country, sample size, study center number, metastatic status, treatment, cut-off value, or type of survival analysis (**Table 3**).

**Table 3:** Subgroup analysis of prognostic value of AGR for PFS in patients with RCC.

Variables	No. of studies	No. of patients	Effects model	HR (95%CI)	p	Heterogeneity I^2^(%) Ph	
Total	6	4,511	Random	2.44 (1.61-3.70)	<0.001	75.9	0.001
Country							
China	2	603	Fixed	8.00 (4.06-15.75)	<0.001	0	0.676
Other than China	4	3,908	Fixed	1.70 (1.44-2.01)	<0.001	0	0.647
Sample size							
<200	3	512	Fixed	2.05 (1.45-2.89)	<0.001	46.9	0.152
≥200	3	3,999	Random	2.51 (1.36-4.65)	0.003	87.9	<0.001
Study center							
Single center	4	928	Random	3.96 (1.48-10.59)	0.006	78.8	0.003
Multicenter	2	3,583	Fixed	1.66 (1.38-2.00)	<0.001	23.6	0.253
Metastatic status							
Non-metastatic	3	3,548	Random	3.46 (1.07-11.26)	0.039	83.3	0.003
Metastatic	2	776	Fixed	1.61 (1.32-1.97)	<0.001	0	0.396
Mixed	1	187	–	6.45 (1.91-21.80)	0.003	–	–
Treatment							
Surgery	5	4,348	Random	2.84 (1.62-4.98)	<0.001	80.7	<0.001
Targeted therapy	1	163	–	1.84 (1.27-2.65)	0.001	–	–
Cut-off value							
<1.43	3	741	Random	3.43 (1.03-11.40)	0.044	83.0	0.003
≥1.43	3	3,770	Random	1.91 (1.28-2.83)	0.001	66.5	0.050
Survival analysis							
Univariate	1	187	–	6.45 (1.91-21.80)	0.003	–	–
Multivariate	5	4,324	Random	2.21 (1.47-3.30)	<0.001	75.9	0.002

### Relationship between the AGR and clinicopathological features

**Figure 4:**
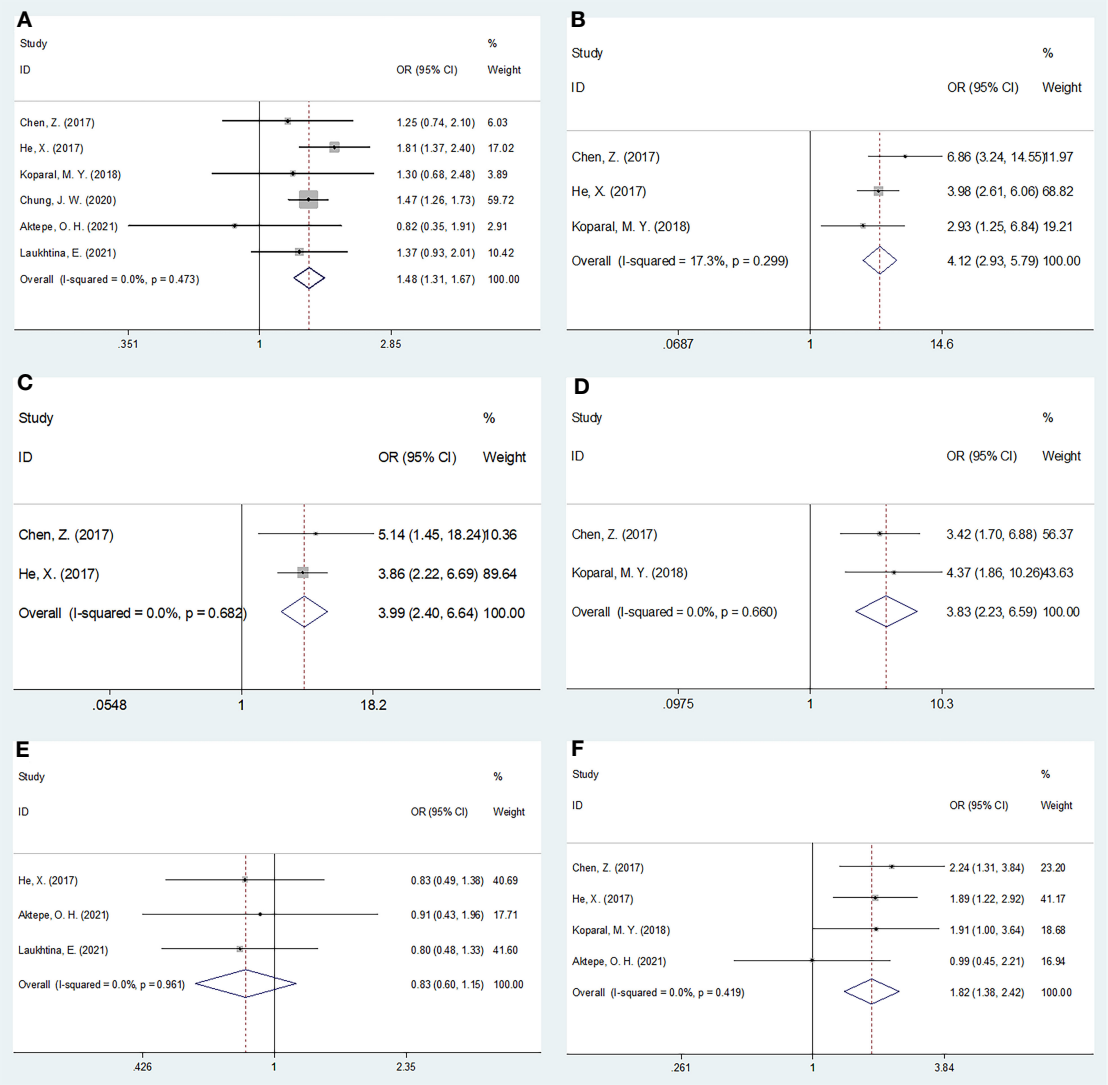
Forest plots evaluating the association between AGR and clinicopathological features in RCC. **(A)** Gender (female vs male); **(B)** pT stage (T3-T4 vs T1-T2); **(C)** pN stage (N1 vs N0); **(D)** Tumor necrosis (present vs absent); **(E)** Histology (ccRCC vs non-ccRCC); and **(F)** Fuhrman grade (G3-G4 vs G1-G2).

The relationship between the pretreatment AGR and the clinicopathological features of RCC was analyzed using data from six studies with 5,219 patients (19–21, 23, 26, 27). The clinicopathological characteristics examined were as follows: sex (female vs male), pT stage (T3–T4 vs T1–T2), pN stage (N1 vs N0), tumor necrosis (present vs absent), histology (clear cell RCC vs non-clear cell RCC), and Fuhrman grade (3–4 vs 1–2). As shown in **Table 4** and **Figure 4**, a low AGR closely correlated with the female sex (OR = 1.48, 95% CI = 1.31–1.67, p <0.001), pT stage T3–T4 (OR = 4.12, 95% CI = 2.93–5.79, p <0.001), pN stage N1 (OR = 3.99, 95% CI = 2.40–6.64, p <0.001), presence of tumor necrosis (OR = 3.83, 95% CI = 2.23–6.59, p <0.001), and Fuhrman grade 3–4 (OR = 1.82, 95% CI = 1.34–2.42, p <0.001). The AGR was not related to histology (OR = 0.83, 95% CI = 0.60–1.15, p = 0.267).

**Table 4:** The association between AGR and clinicopathological features in patients with RCC.

Factors	No. of studies	No. of patients	Effects model	OR (95%CI)	p	Heterogeneity I^2^(%) Ph	
Gender (female vs male)	6	5,219	Fixed	1.48 (1.31-1.67)	<0.001	0	0.473
pT stage (T3-T4 vs T1-T2)	3	1,473	Fixed	4.12 (2.93-5.79)	<0.001	17.3	0.299
pN stage (N1 vs N0)	2	1,311	Fixed	3.99 (2.40-6.64)	<0.001	0	0.682
Tumor necrosis (present vs absent)	2	578	Fixed	3.83 (2.23-6.59)	<0.001	0	0.660
Histology (ccRCC vs non-ccRCC)	3	1,671	Fixed	0.83 (0.60-1.15)	0.267	0	0.961
Fuhrman grade (G3-G4 vs G1-G2)	4	1,636	Fixed	1.82 (1.38-2.42)	<0.001	0	0.419

ccRCC, clear cell renal cell carcinoma.

### Publication bias

**Figure 5:**
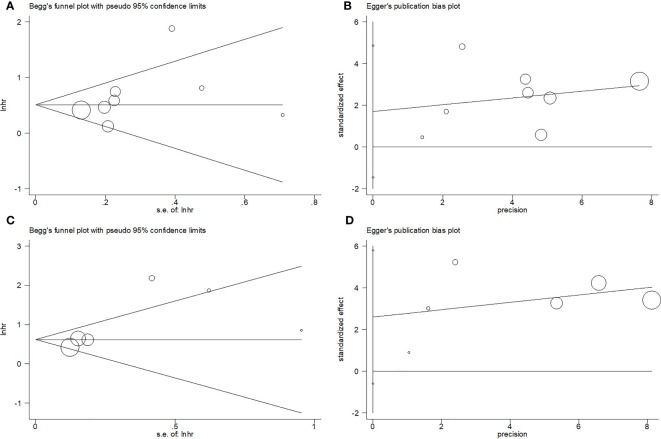
Publication test by Begg’ test and Egger’s test. **(A)** Begg’s test for OS, p=0.266; **(B)** Egger’s test for OS, p=0.236; **(C)** Begg’s test for PFS, p=0.260; and **(D)** Egger’s test for PFS, p=0.087.

Begg’s and Egger’s tests were used to assess publication bias. Funnel plots revealed rough symmetry regarding the distribution of many of the included articles, indicating the absence of obvious publication bias for OS (p = 0.266 and 0.236 for Begg’s and Egger’s tests, respectively) and PFS (p = 0.260 and 0.087, respectively; **Figure 5**).

## Discussion

Whether the AGR predicts RCC prognosis is unclear, with previous reports providing conflicting results (19–27). The present meta-analysis, which synthesized data from nine studies with 5,671 RCC cases, revealed a robust association between a low pretreatment AGR and shorter OS and PFS times. As a reflection of the aggressive nature of RCC, a low AGR also correlated with pT stage T3–T4, pN stage N1, tumor necrosis, and Fuhrman grade 3–4. Hence, it might serve as a stable predictor of the short- and long-term prognosis of patients with RCC; notably, patients with low AGRs tended to experience tumor progression and metastasis. To our knowledge, this is the first reported meta-analysis of the significance of the AGR in predicting the prognosis and clinicopathological features of RCC.

The AGR compares ALB and GLB levels, with a low AGR indicating a low ALB and/or high GLB level. The potential mechanisms underlying the correspondence between a low AGR and poor RCC prognosis reflect the anti-oncogenic and pro-oncogenic actions of ALB and GLB, respectively. Serum ALB, a liver-generated soluble protein, maintains capillary osmotic pressure, removes free radicals from the blood (13), inhibits systemic inflammatory reactions, and serves as a marker of nutritional status (34). Malnutrition and inflammation impede its synthesis, as does interleukin-6 during the acute phase of inflammation in hepatocytes (35). ALB levels have been useful for predicting the outcomes of various cancers (36). The GLB component of the AGR includes diverse proinflammatory proteins, such as immunoglobulins, C-reactive protein, complement components (37), α-2 macroglobulin, fibrinogen, prothrombin, and serum amyloid A (38, 39). Because immunoglobulins are primarily metabolized in the liver, their clearance is impaired in patients with severe hepatic dysfunction, resulting in hyperglobulinemia (40, 41). In support of a potential role of GLB in apoptosis and carcinogenesis, a previous study associated increases in immunoglobulin levels with variations in the gene encoding tumor necrosis factor receptor 13B (42). Therefore, the AGR, which considers both ALB and GLB levels, is a reasonable and reliable prognostic marker.

Notably, a recent large-scale multicenter real-world retrospective study used pretreatment clinical characteristics to identify patients with muscle-invasive bladder cancer (MIBC) who could benefit from neoadjuvant combination therapy (43). Based on the combined prognostic efficiency of four hematological indexes [platelet-to-lymphocyte ratio (PLR) and hemoglobin, globulin, and platelet levels], a new indicator, the PLR.GHR (PLR*Globulin/Hemoglobin), was developed for MIBC (43). This finding has important implications in terms of the findings of our meta-analysis on RCC

Previous studies have evaluated various hematological parameters as predictors of RCC outcomes. In the report by De Giorgi et al., the systemic immune inflammation index and body mass index independently predicted OS in RCC patients treated with nivolumab (44). In studies of mRCC, a low pretreatment prognostic nutritional index was a potential risk factor after first-line therapy with tyrosine kinase inhibitors (45), and the PLR was an independent indicator of survival in a large cohort (n = 921) (46). Additionally, a recent meta-analysis associated a high pretreatment neutrophil-to-lymphocyte ratio and PLR with progression and mortality in mRCC patients treated with immune checkpoint inhibitors. Collectively, these studies implicate multiple hematological parameters in RCC prognosis (44–47).

Both units of the AGR (ALB and GLB) are important components of the tumor microenvironment. Therefore, the prognostic capability of the AGR can be influenced by tumor immune markers, such as branched chain aminotransferase 2 (BCAT2) (48), 5 methylcytosine (5mC) (49), and Siglec15 (50), all of which have been shown to shape the tumor microenvironment (48–50). The relationship between the AGR and these markers should be investigated in future studies.

Several meta-analyses suggest that a low pretreatment AGR can predict the prognosis of various cancers (51–55). In a meta-analysis of 3,211 patients with head and neck cancer, a low AGR significantly correlated with poor disease-free survival (DFS), distant metastasis-free survival, OS, T3–T4 status, lymph node metastasis, and stage III–IV disease (51). In a meta-analysis of 7,211 patients with metastatic prostate cancer, the AGR independently predicted PFS and CSS (52). Furthermore, in a meta-analysis of 8,397 patients with colorectal cancer, a low AGR robustly predicted poor OS and DFS/PFS (53). In additional meta-analyses, a low AGR remarkably predicted poor OS and DFS in patients (n = 3,496) with lung cancer (54) and was closely associated with poor OS and DFS/PFS in patients with gastric cancer (12 articles) (55).

The present study had some limitations. First, because all included articles were retrospective, inherent heterogeneity existed. However, the appropriate model for analysis of heterogeneous data (the random-effects model) was used. Second, the sample size was relatively small. Although we searched several databases, only nine relevant studies were retrieved. Third, most of the eligible studies were conducted in Asia. Consequently, our findings might be more applicable to Asian vs non-Asian patients with RCC. Owing to these limitations, larger, multi-arm prospective studies are needed to validate the prognostic significance of the pretreatment AGR in patients with RCC.

In conclusion, a low AGR markedly predicted poor OS and PFS in patients with RCC and significantly correlated with clinicopathological features indicative of disease progression. Use of the AGR will aid the identification of high-risk individuals and expedite the development of effective therapeutic strategies for RCC.

## Data availability statement

The data that support the findings of this study are available from the corresponding author upon reasonable request.

## Author contributions

HM and FY contributed to conception and design of the study. HM and FY were in charge of data collection. HM and FY performed the statistical analysis. HM wrote the first draft of the manuscript. FY edited the manuscript. All authors contributed to the article and approved the submitted version.
